# Prevalence of hyperthyroidism and hypothyroidism in liver transplant recipients and associated risk factors

**DOI:** 10.1038/s41598-024-58544-3

**Published:** 2024-04-03

**Authors:** Moises Alberto Suarez-Zdunek, Nicoline Stender Arentoft, Paul Suno Krohn, Emilie Høegholm Ernst Lauridsen, Shoaib Afzal, Julie Høgh, Magda Teresa Thomsen, Andreas Dehlbæk Knudsen, Børge Grønne Nordestgaard, Jens Georg Hillingsø, Gerda Elisabeth Villadsen, Peter Holland-Fischer, Allan Rasmussen, Anette Dam Fialla, Ulla Feldt-Rasmussen, Susanne D. Nielsen

**Affiliations:** 1 Department of Infectious Diseases, Copenhagen University Hospital – Rigshospitalet, Esther Møllers Vej 6, 2100 Copenhagen, Denmark; 2 Department of Surgical Gastroenterology, Copenhagen University Hospital – Rigshospitalet, Copenhagen, Denmark; 3 Department of Hepatology and Gastroenterology, Aarhus University Hospital, Aarhus, Denmark; 4 Department of Clinical Biochemistry, Copenhagen University Hospital – Herlev and Gentofte, Herlev, Denmark; 5 Copenhagen General Population Study, Copenhagen University Hospital – Herlev and Gentofte, Herlev, Denmark; 6 Department of Clinical Medicine, Faculty of Health and Medical Sciences, University of Copenhagen, Copenhagen, Denmark; 7 Department of Gastroenterology, Aalborg University Hospital, Aalborg, Denmark; 8 Department of Gastroenterology, Odense University Hospital, Odense, Denmark; 9 Department Endocrinology and Metabolism, Copenhagen University Hospital – Rigshospitalet, Copenhagen, Denmark

**Keywords:** Epidemiology, Liver, Thyroid diseases

## Abstract

The prevalence of hyperthyroidism and hypothyroidism and associated risk factors are unknown in liver transplant recipients. We aimed to determine the prevalence of hyperthyroidism and hypothyroidism and associated risk factors in liver transplant recipients and to compare it with controls from the general population. As part of the Danish Comorbidity in Liver Transplant Recipients (DACOLT) Study, all Danish liver transplant recipients over the age of 20 were invited for measurements of concentrations of thyrotropin and thyroid hormones. The prevalence of hyperthyroidism and hypothyroidism was compared to age- and sex-matched controls from the Copenhagen General Population Study. Using logistic regression adjusted for age, sex, smoking, and body-mass index, we investigated potential risk factors. We recruited 489 liver transplant recipients and 1808 controls. Among liver transplant recipients, 14 (2.9%) had hyperthyroidism compared with 21 (1.2%) of controls (adjusted odds ratio [aOR] 2.24, 95% confidence interval [CI] 1.05–4.75, *P* = 0.04), while 42 (5.7%) had hypothyroidism compared with 139 (7.7%) of controls (aOR 0.68, 95% CI 0.43–1.08, *P* = 0.10). Female sex, and autoimmune hepatitis and primary sclerosing cholangitis as causes of transplantation were associated with hyperthyroidism after adjustments. Age, female sex, and autoimmune liver diseases as cause of transplantation were associated with hypothyroidism after adjustments. DACOLT is registered in ClinicalTrials.gov (NCT04777032).

## Introduction

Liver transplantation is a life-saving procedure for a wide variety of end-stage liver diseases^1^. The distribution of causes of transplantation varies worldwide, but autoimmune liver diseases are important indications for liver transplantation^2^. Many liver transplant recipients live to experience non-hepatic comorbidities^3^, and identifying non-hepatic comorbidities may potentially improve outcomes and quality of life for patients living with a transplanted liver. Thyroid dysfunction includes hyperthyroidism and hypothyroidism and is due to inappropriate secretion and metabolism of thyroid hormones. The liver is involved in normal thyroid hormone metabolism as well as in the abnormal metabolism seen in non-thyroidal illness^4–6^. Furthermore, autoimmune liver diseases are also associated with autoimmune thyroid disease (AITD)^7^. However, only few studies with small sample sizes, highly selected populations and lack of non-transplanted controls have examined thyroid dysfunction after liver transplantation with conflicting findings^8,9^. Thus, it is unknown if hyperthyroidism or hypothyroidism are comorbidities of particular concern in liver transplant recipients.

The aim of this study was to determine the prevalence of hyperthyroidism and hypothyroidism in liver transplant recipients and compare it to age- and sex-matched controls. In addition, we aimed to determine risk factors associated with hyperthyroidism and hypothyroidism in liver transplant recipients. We hypothesise that there is a higher prevalence of hyperthyroidism and hypothyroidism among liver transplant recipients than in controls and that transplantation-related variables, including causes of transplantation and immunosuppressants, are associated with hyperthyroidism and hypothyroidism.

## Methods

### Overview

The Danish Comorbidity in Liver Transplant Recipients (DACOLT) Study (ClinicalTrials.gov: NCT04777032) is a nation-wide, prospective, longitudinal cohort study that aims to determine the burden of comorbidities in liver transplant recipients. All Danish liver transplant recipients aged 20 or more have been invited, and > 85% have chosen to participate. The protocol has been published elsewhere^10^. The Copenhagen General Population Study (CGPS) is a prospective, longitudinal cohort study that recruits adults over the age of 20 from the general population in the Copenhagen area in Denmark^11^. For this cross-sectional study, we recruited liver transplant recipients from DACOLT and general population controls from CGPS who had available measurements of blood concentrations of thyrotropin (TSH), total thyroxine (tT4), total triiodothyronine (tT3) and free thyroxine (fT4). Controls were frequency matched on sex and age with a maximal time interval between examinations of five years.

### Ethics declaration

The study was conducted in accordance with the Declaration of Helsinki. The DACOLT and CGPS studies have been approved by the Ethics Committee of the Capital Region of Denmark (approvals H-20052199 and H-KF-01-144/01). Oral and written informed consent was obtained from all subjects. All liver transplantations in Denmark are performed at the Copenhagen University Hospital – Rigshospitalet with prior consent from donors and recipients, and organs are not procured from prisoners.

### Data acquisition

At inclusion, all participants in DACOLT and CGPS completed a questionnaire on smoking, alcohol intake, medical history and use of thyroid medications and antidiabetics, and they underwent a physical examination, including measurement of weight and height, performed by trained personnel.

Venous blood samples were collected between 2021 and 2023 from liver transplant recipients and between 2016 and 2017 from controls, and concentrations of TSH, tT4, tT3, and fT4 were measured at the laboratory of the hospital at which the sample was collected.

For liver transplant recipients, patient records were reviewed for the cause of liver transplantation, time since first liver transplantation, re-transplantation, and history of type 1 diabetes mellitus (T1DM). History of inflammatory bowel disease, coeliac disease, rheumatoid arthritis, psoriasis, multiple sclerosis, and/or any connective tissue disease was retrieved from the questionnaire. Data on use of immunosuppressants in liver transplant recipients at the time of inclusion were collected from the Shared Medication Record (FMK), which includes all prescribed out-patient medications in Denmark. Results of severe acute respiratory syndrome coronavirus 2 (SARS-CoV-2) tests were retrieved from the Danish Microbiology Database that contains complete data from all departments of clinical microbiology in Denmark since 2010, including results of all SARS-CoV-2 polymerase chain reaction (PCR) and antigen tests from public test centres^12^.

### Definitions

Hyperthyroidism was defined as TSH concentration below reference range or self-reported use of medications for hyperthyroidism irrespective of TSH concentration. Hypothyroidism was defined as TSH concentration above reference range or self-reported use of medications for hypothyroidism irrespective of TSH concentration. Euthyroidism was defined as TSH concentration within reference range and no self-reported use of medications for either hyperthyroidism or hypothyroidism.

For participants who were not treated with thyroid medications, overt hyperthyroidism was defined as TSH concentration below reference range and fT4 and/or tT3 concentrations above reference ranges, while subclinical hyperthyroidism was defined as TSH concentration below reference range and fT4 and tT3 concentrations within reference ranges. Overt hypothyroidism was defined as TSH concentration above reference range and fT4 below reference range, while subclinical hypothyroidism was defined as TSH concentration above reference range and fT4 concentration within reference range. Biochemical euthyroidism was defined as TSH concentration within reference range.

Assays and reference ranges of the analysing laboratories are provided in the Supplementary Table S1.

Ethnicity was based on the origin of the participants’ grandparents as reported by the participant. T1DM was defined as use of insulin for a recorded T1DM diagnosis in the medical record. BMI categories were defined in accordance with WHO definitions^13^: Underweight (< 18.5 kg/m^2^), normal weight (18.5–24.9 kg/m^2^), overweight (25.0–29.9 kg/m^2^), and obese (≥ 30.0 kg/m^2^). Smoking status was defined as either current, former, or never smoker, while cumulative smoking was defined as pack-years (years smoking 20 cigarettes per day) in former or current smokers. Autoimmune liver disease was defined as autoimmune hepatitis (AIH), primary biliary cholangitis (PBC), primary sclerosing cholangitis (PSC) and/or other autoimmune liver disease as causes of liver transplantation. Non-thyroidal autoimmune disease was defined as either autoimmune liver disease, T1DM, any inflammatory bowel disease, coeliac disease, rheumatoid arthritis, psoriasis, multiple sclerosis, and/or any connective tissue disease. History of coronavirus disease 2019 (COVID-19) was defined as any positive SARS-CoV-2 test prior to date of examination.

### Statistical analyses

For univariable and multivariable analyses, we used univariable and multivariable logistic regressions to calculate the odds ratios (OR) and adjusted odds ratios (aOR) of each variable of interest, respectively. Participants with missing data were omitted in the regression models. Model assumptions were assessed graphically. The outcome was hyperthyroidism or hypothyroidism. The additional classification in overt and subclinical hyperthyroidism and hypothyroidism was used for descriptive statistics but not in the regression models.

An adjusted base model was defined a priori and included age, sex, smoking status, and BMI. Age, sex, smoking status, cumulative smoking, BMI, ethnicity, previous re-transplantation, cause of transplantation, time since first liver transplantation, use of calcineurin inhibitor at inclusion, use of antimetabolite at inclusion, use of azathioprine at inclusion, use of prednisolone at inclusion, and use of everolimus at inclusion were tested one at a time in liver transplant recipients before and after adjustment for the base model. Use of calcineurin inhibitor at inclusion was treated as a categorical variable: tacrolimus, ciclosporin, or none of these at inclusion. Use of antimetabolite at inclusion was also treated as a categorical variable: mycophenolic acid/mycophenolate mofetil (MPA/MMF), azathioprine, or none of these at inclusion. Afterwards, adjusted for the base model, liver transplantation status and interactions between liver transplantation status and age, sex, smoking status, cumulative smoking, BMI, and ethnicity were tested one at a time.

A priori, we planned three sensitivity analyses. First, excluding liver transplant recipients transplanted < 6 months prior to inclusion. Second, excluding liver transplant recipients with non-thyroidal autoimmune disease. Third, we tested if analysing laboratory or out-patient centre were associated with hyperthyroidism or hypothyroidism in liver transplant recipients. Post hoc, we performed two sensitivity analyses. First, we excluded all participants with history of COVID-19. Second, we additionally adjusted the base model for ethnicity.

Two-sided *p*-values of 0.05 or less were deemed statistically significant. For all analyses, we used R (version 1.2.5001) with the *sandwich* package (version 3.0-1)^14^.

## Results

We included 489 liver transplant recipients and 1808 age- and sex-matched controls. Among liver transplant recipients and controls, 54% were male, and the median age was 55.3 (interquartile range [IQR] 45.8–63.8) in liver transplant recipients and 56.5 (IQR 47.7–64.8) in controls. While smoking habits were similar, fewer liver transplant recipients than controls had current intake of alcohol, normal weight, and Scandinavian ethnicity (Table 1). The most common cause of liver transplantation was autoimmune liver disease (43%), followed by alcoholic or cryptogenic cirrhosis (18%) (Table 2).

**Table 1:** Characteristics of liver transplant recipients and controls (*N* = 2297).

	LT recipients (*n* = 489)	Controls (*n* = 1808)
Age, years, median (IQR)	55.3 (45.8–63.8)	56.5 (47.7–64.8)
Male sex, n (%)	266 (54%)	972 (54%)
Smoking, pack years, median (IQR)	0 (0–9)	0 (0–11.25)
Current	61 (12%)	199 (11%)
Former	159 (33%)	700 (39%)
Never	257 (53%)	909 (50%)
Missing	12 (2.5%)	0 (0%)
BMI, kg/m^2^, median (IQR)	26.7 (23.7–30.0)	25.3 (23.1–28.4)
Underweight, n (%)	6 (1.2%)	20 (1.1%)
Normal, n (%)	158 (32.3%)	822 (45.5%)
Overweight, n (%)	196 (40.1%)	666 (36.8%)
Obese, n (%)	120 (24.5%)	296 (16.4%)
Missing	9 (1.8%)	4 (0.2%)
Ethnicity, n (%)		
Scandinavian	393 (80.4%)	1666 (92.1%)
Other European	48 (9.8%)	110 (6.1%)
Middle East and Indian Subcontinent	23 (4.7%)	2 (0.1%)
Other	16 (3.3%)	14 (0.8%)
Missing	9 (1.8%)	16 (0.9%)
Alcohol intake, units per week, n (%)		
0	250 (51.1%)	218 (12.1%)
0.1–7	166 (34.0%)	744 (41.2%)
7.1–14	36 (7.4%)	480 (26.5%)
14.1–21	10 (2.0%)	205 (11.3%)
> 21	8 (1.6%)	156 (8.6%)
Missing	19 (3.9%)	5 (0.2%)

BMI, body mass index: underweight (< 18.5), normal (18.5–24.9), overweight (25.0–25.9), obese (≥ 30.0); Ethnicity, origin of parents and grandparents; IQR, inter-quartile range; LT, liver transplant.

**Table 2:** Transplantation-related characteristics of liver transplant recipients.

Primary cause of liver transplantation	
Autoimmune liver disease	211 (43.1%)
Autoimmune hepatitis	55 (11.2%)
Primary sclerosing cholangitis	134 (27.4%)
Primary biliary cholangitis	44 (9.0%)
Other autoimmune	10 (2.0%)
Alcoholic or cryptogenic cirrhosis	86 (17.6%)
Hepatocellular carcinoma	38 (7.8%)
Fulminant hepatic failure	32 (6.5%)
Metabolic liver disease	21 (4.3%)
Hepatitis C virus	14 (2.9%)
Other	103 (21.1%)
Out-patient transplantation centre	
Copenhagen University Hospital—Rigshospitalet	276 (56.4%)
Aarhus University Hospital	116 (23.7%)
Odense University Hospital	72 (14.7%)
Aalborg University Hospital	25 (5.1%)
Time since first transplantation, years (IQR)	6.7 (2.8–13.4)
Previous retransplantation	35 (7.2%)
Current use of calcineurin inhibitor	
Tacrolimus	397 (81.2%)
Ciclosporin	49 (10.0%)
None of the above	43 (8.8%)
Current use of antimetabolite	
MPA/MMF	342 (69.9%)
Azathioprine	52 (10.6%)
None of the above	95 (19.4%)
Current use of prednisolone	230 (47.0%)
Current use of everolimus	38 (7.8%)

IQR, inter-quartile range; MPA/MMF, mycophenolic acid/mycophenolate mofetil.

### Associations with liver transplantation status

Hyperthyroidism was present in 14 (2.9%) liver transplant recipients and 21 (1.2%) controls (odds ratio [OR] 2.51 [95% confidence interval 1.27–4.97], *P* < 0.02). Liver transplantation remained significantly associated with hyperthyroidism after adjustment for age, sex, smoking, and BMI (adjusted OR [aOR] 2.24 [1.05–4.75], *P* = 0.04). Hypothyroidism was present in 28 (5.7%) liver transplant recipients and 139 (7.7%) controls (OR 0.73 [0.48–1.11], *P* = 0.14), and liver transplantation was not significantly associated with hypothyroidism in the adjusted model (aOR 0.68 [0.43–1.08], *P* = 0.10). Of liver transplant recipients who had hyperthyroidism and hypothyroidism, 42% and 61% were treated with thyroid medications, respectively (Table 3).

**Table 3:** Thyroid function in liver transplant recipients and controls.

Clinical classification	LT recipients (n = 489)	Controls (n = 1808)
Euthyroidism	447 (91.4%)	1652 (91.4%)
Hyperthyroidism	14 (2.9%)	21 (1.2%)
Use of medications for hyperthyroidism	6 (42.9%)	9 (42.9%)
Hypothyroidism	28 (5.7%)	139 (7.7%)
Use of medications for hypothyroidism	17 (60.7%)	67 (48.2%)

Biochemical classification^a^	LT recipients (n = 466)	Controls (n = 1732)
Euthyroidism	447 (95.9%)	1652 (95.2%)
Hyperthyroidism	8 (1.7%)	12 (0.7%)
Overt hyperthyroidism	0 (0%)	2 (16. 7%)
Subclinical hyperthyroidism	8 (100%)	10 (83.3%)
Hypothyroidism	11 (2.4%)	22 (1.3%)
Overt hypothyroidism	1 (9.1%)	21 (54.5%)
Subclinical hypothyroidism	10 (90.9%)	10 (45.5%)

LT, liver transplant.

^a^For the biochemical classification, only participants not using thyroid medications were included.

### Associations with demographic variables and lifestyle

Female sex was associated with hyperthyroidism before (OR 4.55 [1.25–16.5], *P* = 0.02) and after adjustments (aOR 4.50 [1.21–16.7], *P* = 0.03), while the association between age and hyperthyroidism was borderline significant before (OR 1.56 [1.00–2.42] per decade, *P* = 0.051) and after adjustments (aOR 1.51 [0.95–2.37] per decade, *P* = 0.08). Smoking status, cumulative smoking, and BMI were not associated with hyperthyroidism (Table 4).

**Table 4:** Associations of potential demographic variables and lifestyle with hyperthyroidism in liver transplant recipients.

	OR (95% CI), *p*	aOR (95% CI), *P*
Age, per decade	1.56 (1.00–2.42), 0.05	1.51 (0.95–2.37), 0.08
Female sex	4.55 (1.25–16.5), **0.02**	4.50 (1.21–16.7), **0.03**
Smoking, per 10 pack-years	1.02 (0.85–1.24), 0.81	0.99 (0.72–1.38), 0.97
Never	Reference	Reference
Current	1.42 (0.28–7.20), 0.42	1.13 (0.21–6.22), 0.84
Former	1.64 (0.52–5.18), 0.84	1.13 (0.34–3.73), 0.89
BMI		
Underweight	NA	NA
Normal	Reference	Reference
Overweight	0.60 (0.13–2.71), 0.51	0.72 (0.16–3.36), 0.68
Obese	2.03 (0.56–7.35), 0.28	2.26 (0.60–8.44), 0.23
Ethnicity		
Scandinavian	Reference	Reference
Other European	0.74 (0.09–5.85), 0.77	0.69 (0.08–5.95), 0.74
Middle East & Indian Subcontinent	1.58 (0.19–12.8), 0.67	3.88 (0.41–36.9), 0.24
Other	2.32 (0.28–19.1), 0.44	3.81 (0.40–35.8), 0.24

aOR, odds ratio adjusted for age, sex, smoking, and BMI; BMI, body-mass index: underweight (< 18.5), normal (18.5–24.9), overweight (25.0–25.9), obese (≥ 30.0); CI, confidence interval; NA, not available due to low number of cases; OR, odds ratio.

Significant values are in [bold]

Age (OR 1.94 [1.36–2.75] per decade, *P* < 0.001), female sex (OR 2.25 [1.02–4.98], *P* = 0.046), and other European compared to Scandinavian ethnicity (OR 5.10 [2.13–12.2], *P* < 0.001) were associated with hypothyroidism. Age (aOR 1.96 [1.36–2.83] per decade, *P* < 0.001), female sex (aOR 2.49 [1.07–5.82], *P* = 0.04), and other European compared to Scandinavian ethnicity (aOR 8.59 [3.18–23.2], *P* < 0.001) all remained associated with hypothyroidism after adjustment for age, sex, smoking, and BMI. Smoking status, cumulative smoking, and BMI were not associated with hypothyroidism (Supplementary Table S2).

There was a significant interaction between liver transplantation and age regarding hypothyroidism. In liver transplant recipients, the odds of having hypothyroidism per decade higher age were higher than in controls (OR 1.94 [1.36–2.75] vs. 1.17 [1.02–1.34], *p* for interaction = 0.009). For hyperthyroidism, this interaction was not significant (OR 1.28 [0.98–1.66] vs. 1.07 [0.82–1.39], *p* for interaction = 0.08).

### Associations with transplantation-related variables

AIH was associated with hyperthyroidism (OR 3.33 [1.01–11.0], *P* = 0.049) and remained so after adjustment for age, sex, smoking, and BMI (aOR 3.72 [1.02–13.6], *P* = 0.046). PSC was not associated with hyperthyroidism before adjustments (OR 2.03 [0.69–5.97], *P* = 0.20), but was associated with hyperthyroidism after adjustments (aOR 6.23 [1.65–23.5], *P* = 0.007).

Overall, autoimmune liver disease as cause of transplantation was not associated with hypothyroidism (OR 1.82 [0.84–3.93], *P* = 0.13) but became so after adjustments (aOR 2.47 [1.04–5.86], *P* = 0.04). When analysed separately, PBC was associated with hypothyroidism before adjustments (OR 4.72 [1.94–11.5], *P* < 0.001) but not after (aOR 2.50 [0.92–6.80], *P* = 0.07). Previous re-transplantation, other causes of transplantation than those mentioned above, as well as time since first liver transplantation, were not associated with either hyperthyroidism (Table 5) or hypothyroidism (Supplementary Table S2).

**Table 5:** Associations of transplantation-related variables with hyperthyroidism.

	OR (95% CI), *p*	aOR (95% CI), *P*
Previous retransplantation	1.00 (0.13–7.86), > 0.99	1.62 (0.19–13.9), 0.66
Time since first LT, per 10 years	0.97 (0.49–1.91), 0.94	0.83 (0.40–1.72), 0.62
Cause of LT		
Autoimmune liver disease	1.79 (0.61–5.23), 0.29	2.16 (0.64–7.28), 0.21
AIH	3.33 (1.01–10.99), **0.049**	3.72 (1.02–13.6),** 0.046**
PSC	2.03 (0.69–5.97), 0.20	6.23 (1.65–23.5), **0.007**
PBC	0.77 (0.10–6.05), 0.81	0.33 (0.04–2.76, 0.30
Alcoholic or cryptogenic cirrhosis	0.78 (0.17–3.53), 0.74	0.80 (0.15–4.10), 0.79
Hepatocellular carcinoma	0.91 (0.12–7.15), 0.93	0.92 (0.11–7.82), 0.94
Fulminant hepatic failure	NA	NA
Metabolic liver disease	NA	NA
Hepatitis C virus	2.73 (0.33–22.5), 0.35	2.79 (0.26–29.5), 0.40
Other	1.02 (0.28–3.74), 0.97	1.12 (0.29–4.28), 0.87

AIH, autoimmune hepatitis; aOR, odds ratio adjusted for age, sex, smoking, and BMI; CI, confidence interval; LT, liver transplantation; NA, not available due to low number of cases; OR, odds ratio; PBC, primary biliary cholangitis; PSC, primary sclerosing cholangitis.

Significant values are in [bold]

Before adjustments, use of prednisolone was not associated with hyperthyroidism in liver transplant recipients (OR 2.90 [0.90–9.37], *P* = 0.08) but became significant after adjustment for age, sex, smoking, and BMI (aOR 3.50 [1.03–11.9], *P* = 0.04), and after a further adjustment for time since first liver transplantation (aOR 3.45 [1.01–11.8], *P* = 0.048). Use of calcineurin inhibitor and use of antimetabolite were not associated with hyperthyroidism or hypothyroidism before or after adjustments.

### Sensitivity analyses

Excluding liver transplant recipients who have been transplanted less than 6 months prior to inclusion did not change any results.

In a sensitivity analysis excluding liver transplant recipients with non-thyroidal autoimmune disease, the parameter estimate of liver transplantation status did not change for hyperthyroidism but became non-significant (OR 2.49 [0.88–7.07], *P* = 0.09). For hypothyroidism, the parameter estimate of liver transplantation status became borderline significant (OR 0.54 [0.28–1.03], *P* = 0.06). Furthermore, results did not differ from the primary analyses for sex, smoking status, cumulative smoking, BMI, and ethnicity. However, the association between hyperthyroidism and age did not reach significance (aOR 2.10 [0.83–5.26], *P* = 0.12).

In an analysis adjusted for age, sex, smoking, and BMI, analysing laboratory and out-patient centre were not associated with hyperthyroidism or hypothyroidism.

In total, 118 (24%) liver transplant recipients had a history of COVID-19 prior to inclusion and were excluded in a post hoc sensitivity analysis. Liver transplantation status remained associated with hyperthyroidism before (OR 3.13 [1.49–6.57], *P* < 0.01) and after (aOR 3.04 [1.41–6.53], *P* < 0.01) adjusting for age, sex, smoking, and BMI. The estimates for hypothyroidism remained unchanged before (OR 0.77 [0.48–1.24], *P* = 0.29) and after adjusting for age, sex, smoking, and BMI (aOR 0.78 [0.47–1.28], 0.32). All results of this sensitivity analysis are shown in Supplementary Tables S3,S4.

In another post hoc sensitivity analysis adjusting for age, sex, smoking, BMI and ethnicity, liver transplantation status remained associated with hyperthyroidism (aOR 2.37 [1.15–4.87], *P* = 0.02), while there was still no significant association with hypothyroidism (aOR 0.72 [0.46–1.13], *P* = 0.15).

## Discussion

In this nation-wide cross-sectional study of all Danish liver transplant recipients, we found that liver transplant recipients have twice the odds of having hyperthyroidism compared to age- and sex matched controls from the general population independently of age, sex, smoking, and BMI, but similar odds of hypothyroidism. All untreated participants with hyperthyroidism had subclinical disease. This finding is clinically important, as in the general population, even subclinical hyperthyroidism requires monitoring and is associated with higher risk of cardiovascular morbidity, fractures, and overall mortality in older adults^15^.

All but one of liver transplant recipients with untreated hypothyroidism had subclinical disease. With current guidelines, treatment is not necessarily indicated for subclinical hypothyroidism^16^, but hypothyroidism, including subclinical hypothyroidism, before liver transplantation for hepatocellular carcinoma is associated with poorer survival^17,18^. Among liver transplant recipients, we found a combined prevalence of hyperthyroidism and hypothyroidism of 8.7%, which differs from two Brazilian studies of highly selected participants transplanted mainly for cirrhosis after hepatitis C virus (HCV) infection or alcohol use. The first study included 30 liver transplant recipients and found no cases of thyroid dysfunction 6 months after transplantation^9^, while the other study included 46 liver transplant recipients with type 2 diabetes mellitus and found a prevalence of 13%^8^. Significantly smaller sample size, selection based on specific comorbidities, and different causes of transplantation in the previous studies may explain the differences.

In this study, we saw that increasing age and female sex were important risk factors associated with both hyperthyroidism and hypothyroidism, while other European ethnicity than Scandinavian was associated with hypothyroidism. One reference range may not sufficiently encompass physiological differences among euthyroid persons of different ethnicities^19^, but the finding is in line with the Rotterdam Study of the general population that found that age, sex, and genetic predisposition were important risk factors associated with thyroid function^20^. In the Rotterdam Study, TSH concentrations decreased in smokers and increased with higher BMI. Although we could not compare TSH concentrations directly across laboratories due to different reference values at different study sites, we did not find an association between hyperthyroidism or hypothyroidism and either smoking or BMI, indicating that these factors are less prominent in hyperthyroidism or hypothyroidism in liver transplant recipients than age, sex, and ethnicity. For lifestyle variables, missing data was < 2.5% and is unlikely to affect the results.

Morphologic alterations of the thyroid and non-thyroidal illness syndrome are common in severe liver disease^21,22^. Thyroid hormone levels may normalise after liver transplantation^23,24^, but the long-term thyroid function in liver transplant recipients is poorly described. We found evidence that being liver transplanted modifies the effect of age on hypothyroidism, and odds of having hypothyroidism are higher with increasing age among liver transplant recipients than in the general population. This has not previously been reported in liver transplant recipients, but the phenomenon of a chronic condition modifying the effect of age on age-related comorbidities has been reported among, e.g., people with HIV^25^. Overall, the interaction suggests that clinical suspicion of or screening for hyperthyroidism and hypothyroidism in ageing liver transplant recipients should be considered earlier than in the general population, as the long-term effect of subclinical disease is uncertain in liver transplant recipients.

We found that AIH and PSC as causes of transplantation were each associated with hyperthyroidism, while PBC seemed to drive the association between hypothyroidism and autoimmune liver diseases, overall, as causes of transplantation. This is in line with previous studies of the general population where PBC and AIH were associated with hypothyroidism due to AITD^7,26–28^. The association between autoimmune liver diseases and hyperthyroidism is less well explored, but abnormal liver function tests are common in both hyperthyroidism and hypothyroidism, and multiple autoimmune diseases, including autoimmune liver disease and AITD, often coexist^7,29^.

Immunosuppressants may impact on the prevalence and severity of AITD^29^. Calcineurin inhibitors decrease regulatory T cell activity, which has been proposed to promote AITD^30,31^. Standard immunosuppression after liver transplantation in our clinics includes a calcineurin inhibitor, an antimetabolite, and corticosteroids^32^. In this study, we found that use of prednisolone was associated with hyperthyroidism in the adjusted models. This effect is probably due to confounding by indication, as corticosteroids in our clinics are often discontinued a year after transplantation, but frequently continued in recipients with autoimmune disease. We did not find evidence to suggest that other immunosuppressants are associated with hyperthyroidism or hypothyroidism in liver transplant recipients.

As COVID-19 may induce changes in thyroid function, comparing liver transplant recipients included during the pandemic with controls included before the pandemic may potentially impact the results^33,34^. However, access to COVID-19 tests was free and unrestricted for the public in Denmark, and we had access to all test results. As most data were collected during 2021 and early 2022, a minority of liver transplant recipients had a history of COVID-19^35^. Therefore, we were able to exclude participants with history of COVID-19 in a sensitivity analysis, and this did not affect our findings.

Strengths of this study include the large cohort size of around 85% of all liver transplant recipients in Denmark. With matched controls from the general population, investigation of interactions between liver transplantation and possible risk factors are possible. Furthermore, clinical measurements were performed uniformly in liver transplant recipients and in controls. With nation-wide access to hospital records, we also had access to detailed and complete data on transplantation-related variables and immunosuppressants on all liver transplant recipients.

A limitation is the use of different laboratories. Controls are recruited from an area of previous mild iodine deficiency prior to introduction of mandatory iodine fortification in 2000, while some liver transplant recipients live in areas of previous moderate iodine deficiency. After iodine fortification, geographical differences in hyperthyroidism incidence have disappeared by 2006–2007^36^, although there was still a higher incidence of hypothyroidism in areas of mild iodine deficiency by 2008^37^. In this contemporary cohort, we saw no difference in prevalence across the transplantation centres, so geographical differences are unlikely to account for the findings. Furthermore, the ethnic distribution of liver transplant recipients and controls from the capital area differed, but findings were consistent after further adjustment for ethnicity.

As data on autoimmune diseases among controls were not available, we were not able to include non-thyroidal autoimmune diseases in the adjusted models. Furthermore, the number of cases with hyperthyroidism limits the power to investigate potential risk factors. With a cross-sectional design, we cannot determine if the found associations reflect causation, and future longitudinal studies are warranted, preferably including pre- and post-transplantation thyroid function tests to investigate the incidence of de novo hyperthyroidism and hypothyroidism after liver transplantation. The high proportion of autoimmune diseases as causes of transplantation is a limitation to generalisability of this study to areas with a high proportion of other causes, e.g., metabolic dysfunction-associated steatohepatitis or viral hepatitis.

In conclusion, among liver transplant recipients, the prevalence of hyperthyroidism was twice that in age- and sex-matched controls from the general population. Higher clinical suspicion of hyperthyroidism may be warranted in clinics that follow liver transplant recipients as they age.

### Supplementary Information

Supplementary Information.

## Data Availability

The datasets generated during and/or analysed during the current study are not publicly available as they contain information that could compromise participant privacy and consent but are available from the corresponding author on reasonable request and with permission of the transplantation centres.
