# Supplementary material for: Prevalence of hyperthyroidism and hypothyroidism in liver transplant recipients and associated risk factors
Source: Sci Rep. 2024 Apr 3;14:7828. doi: 10.1038/s41598-024-58544-3 (PMC10991542; doi:10.1038/s41598-024-58544-3)
Supplement: Supplementary file 1 — Supplementary Information. [file 41598_2024_58544_MOESM1_ESM.pdf]

Supplementary Table S1 Reference intervals for hormone concentrations

	**TSH** (mIU/L)	**tT4** (nmol/L)	**tT3** (nmol/L)	**fT4** (pmol/L)
Liver transplant recipients				
RH[a]	0.40-4.8	70-140	1.4-2.8	12.0-22.0
AUH[b]	0.30-4.5	60-140	1.1-2.5	12.0-22.0
Aalborg UH[c]	0.30-4.5	60-140	1.1-2.5	10.8-16.8
OUH[a]	0.30-4.0	60-130	1.3-2.2	10.0-22.0
Controls				
HGH[d]	0.40-4.8	70-140	1.0-2.6	11.5-22.7

Aalborg UH, Aalborg University Hospital; **AUH**, Aarhus University Hospital; **fT4**, free thyroxine. **HGH**, Copenhagen University Hospital – Herlev and Gentofte; **OUH**, Odense University Hospital; **RH**, Copenhagen University Hospital – Rigshospitalet; **TSH**, thyrotropin; **tT3**, total triiodothyronine; **tT4**, total thyroxine.

[a]Cobas 8000 e801, Roche, Basel, Switzerland

[b]Atellica IM TSH3-UL, Siemens Healthcare Diagnostics, Tarrytown, NY, USA

[c]Alinity, Abbott Diagnostics, Abbott Park, IL, USA

[d]ADVIA Centaur TSH, Siemens Healthcare Diagnostics, Tarrytown, NY, USA

Supplementary Table S2 Analyses of associations with hypothyroidism in liver transplant recipients

	**OR (95% CI), *P***	**aOR (95% CI), *P***
**Demographic variables and lifestyle**		
Age, per decade	1.94 (1.36-2.75), <**0.001**	1.96 (1.36-2.83), <**0.001**
Female sex	2.25 (1.02-4.98), **0.046**	2.49 (1.07-5.82), **0.04**
Smoking, per 10 pack-years	1.07 (0.97-1.19), 0.20	1.06 (0.95-1.19), 0.30
Never	*Reference*	*Reference*
Current	1.73 (0.52-5.72), 0.37	1.53 (0.44-5.29), 0.50
Former	2.20 (0.94-5.14), 0.07	1.76 (0.73-4.22), 0.21
BMI		
Underweight	NA	NA
Normal	*Reference*	*Reference*
Overweight	0.64 (0.26 1.59), 0.34	0.76 (0.29-1.96), 0.56
Obese	0.95 (0.37 2.45), 0.92	0.86 (0.31-2.39), 0.78
Ethnicity		
Scandinavian	*Reference*	*Reference*
Other European	5.10 (2.13-12.2), <**0.001**	8.59 (3.18-23.2), <**0.001**
Middle East & Indian Subcontinent	1.01 (0.13-7.90), >0.99	2.20 (0.25-19.3), 0.48
Other	1.47 (0.18-11.8), 0.72	2.23 (0.26-19.5), 0.47
**Transplantation-related variables**		
Previous retransplantation	0.47 (0.06-3.53), 0.459	0.55 (0.07-4.34), 0.567
Time since first LT, per 10 years	1.37 (0.88-2.12), 0.159	1.09 (0.67-1.78), 0.728
Cause of LT		
Autoimmune liver disease	1.82 (0.84-3.93), 0.128	2.47 (1.04-5.86), **0.040**
AIH	1.79 (0.65-4.91), 0.260	2.25 (0.76-6.70), 0.144
PSC	0.56 (0.21-1.50), 0.249	1.35 (0.45-4.11), 0.592
PBC	4.72 (1.94-11.5), <**0.001**	2.50 (0.92-6.80), 0.072
Alcoholic or cryptogenic cirrhosis	0.55 (0.16-1.85), 0.332	0.28 (0.06-1.26), 0.097
Hepatocellular carcinoma	0.91 (0.21-3.98), 0.898	0.73 (0.16-3.41), 0.690
Fulminant hepatic failure	0.51 (0.07-3.91), 0.520	0.58 (0.07-4.74), 0.609
Metabolic liver disease	0.82 (0.11-6.32), 0.846	1.26 (0.14-10.9), 0.836
Hepatitis C virus	1.28 (0.16-10.1), 0.817	1.07 (0.12-9.48), 0.952
Other	0.61 (0.21-1.80), 0.369	0.76 (0.25-2.35), 0.635

BMI, body-mass index: underweight (<18.5), normal (18.5-24.9), overweight (25.0-25.9), obese (≥30.0); **LT**, liver transplantation; **AIH**, autoimmune hepatitis; **PSC**, primary sclerosing cholangitis; **PBC**, primary biliary cholangitis; **OR**, odds ratio; **CI**, confidence interval; **NA**, not available due to low number of cases; **aOR**, odds ratio adjusted for age, sex, smoking, and BMI.

Supplementary Table S3 Analyses of associations with hyperthyroidism in liver transplant recipients without history of coronavirus disease 2019

	**OR (95% CI), *P***	**aOR (95% CI), *P***
**Demographic variables and lifestyle**		
Age, per decade	1.51 (0.94-2.43), 0.09	1.44 (0.87-2.36), 0.15
Female sex	7.71 (1.69-35.3), **<0.01**	7.80 (1.67-36.5), <**0.01**
Smoking, per 10 pack-years	1.01 (0.83-1.23), 0.88	0.99 (0.70-1.40), 0.97
Never	*Reference*	*Reference*
Current	1.42 (0.27-7.51), 0.68	1.23 (0.21-7.32), 0.82
Former	1.97 (0.59-6.61), 0.27	1.39 (0.39-4.93), 0.61
BMI		
Underweight	NA	NA
Normal	*Reference*	*Reference*
Overweight	0.82 (0.16-4.12), 0.81	1.05 (0.20-5.47), 0.96
Obese	2.45 (0.60-10.1), 0.21	2.71 (0.63-11.6), 0.18
Ethnicity		
Scandinavian	*Reference*	*Reference*
Other European	0.91 (0.11-7.31), 0.93	0.98 (0.11-8.76), 0.99
Middle East & Indian Subcontinent	1.61 (0.20-13.3), 0.66	5.92 (0.51-68.4), 0.15
Other	2.23 (0.27-18.8), 0.46	3.87 (0.38-39.2), 0.25
**Transplantation-related variables**		
Previous retransplantation	1.02 (0.13-8.16), 0.98	1.67 (0.19-14.9), 0.65
Time since first LT, per 10 years	1.07 (0.53-2.15), 0.85	0.97 (0.47-2.03), 0.95
Cause of LT		
Autoimmune liver disease	1.53 (0.50-4.64), 0.45	1.46 (0.41-5.11), 0.56
AIH	3.09 (0.91-10.5), 0.07	3.03 (0.80-11.4), 0.10
PSC	1.68 (0.54-5.27), 0.37	4.59 (1.16-18.3), **0.03**
PBC	0.95 (0.12-7.53), 0.96	0.29 (0.03-2.58), 0.27
Alcoholic or cryptogenic cirrhosis	0.76 (0.17-3.51), 0.73	0.88 (0.15-5.04), 0.89
Hepatocellular carcinoma	1.27 (0.16-10.2), 0.82	1.20 (0.13-11.3), 0.87
Fulminant hepatic failure	NA	NA
Metabolic liver disease	NA	NA
Hepatitis C virus	2.40 (0.29-20.0), 0.43	3.23 (0.26-39.4), 0.36
Other	1.33 (0.36-4.96), 0.67	1.92 (0.47-7.86), 0.37

BMI, body-mass index: underweight (<18.5), normal (18.5-24.9), overweight (25.0-25.9), obese (≥30.0); **LT**, liver transplantation; **AIH**, autoimmune hepatitis; **PSC**, primary sclerosing cholangitis; **PBC**, primary biliary cholangitis; **OR**, odds ratio; **CI**, confidence interval; **NA**, not available due to low number of cases; **aOR**, odds ratio adjusted for age, sex, smoking, and BMI.

Supplementary Table S4 Analyses of associations with hypothyroidism in liver transplant recipients without history of coronavirus disease 2019

	**OR (95% CI), *P***	**aOR (95% CI), *P***
**Demographic variables and lifestyle**		
Age, per decade	1.82 (1.22-2.73), <**0.01**	1.94 (1.23-3.04), <**0.01**
Female sex	2.43 (0.99-5.95), 0.051	2.87 (1.09-7.58), **0.03**
Smoking, per 10 pack-years	1.08 (0.97-1.19), 0.18	1.07 (0.96-1.20), 0.22
Never	*Reference*	*Reference*
Current	2.94 (0.76-11.4), 0.12	2.74 (0.67-11.2), 0.16
Former	4.17 (1.43-12.2), <**0.01**	3.65 (1.22-11.0), **0.02**
BMI		
Underweight	NA	NA
Normal	*Reference*	*Reference*
Overweight	0.81 (0.28-2.38), 0.70	0.97 (0.31-3.05), 0.96
Obese	1.38 (0.48-3.95), 0.55	1.20 (0.38-3.79), 0.75
Ethnicity		
Scandinavian	*Reference*	*Reference*
Other European	5.94 (2.18-16.2), <**0.001**	9.32 (2.95-29.4), <**0.001**
Middle East & Indian Subcontinent	1.23 (0.15-9.90), 0.85	2.74 (0.28-26.5), 0.38
Other	1.70 (0.21-14.0), 0.62	2.99 (0.32-28.3), 0.34
**Transplantation-related variables**		
Previous retransplantation	NA	NA
Time since first LT, per 10 years	0.99 (0.57-1.74), 0.98	0.79 (0.42-1.48), 0.47
Cause of LT		
Autoimmune liver disease	2.94 (1.17-7.40), **0.02**	4.73 (1.60-13.7), <**0.01**
AIH	2.04 (0.72-5.80), 0.18	2.49 (0.80-7.82), 0.13
PSC	0.76 (0.27-2.13), 0.62	2.20 (0.67-7.17), 0.19
PBC	6.61 (2.45-17.8), <**0.001**	3.04 (0.95-9.68), 0.06
Alcoholic or cryptogenic cirrhosis	0.41 (0.09-1.78), 0.23	0.12 (0.02-1.02), 0.052
Hepatocellular carcinoma	1.56 (0.34-7.13), 0.57	1.27 (0.25-6.55), 0.78
Fulminant hepatic failure	NA	NA
Metabolic liver disease	1.14 (0.14-9.09), 0.90	2.21 (0.23-21.5), 0.50
Hepatitis C virus	1.34 (0.17-10.8), 0.79	1.22 (0.13-11.3), 0.86
Other	0.42 (0.10-1.84), 0.25	0.53 (0.11-2.50), 0.43

BMI, body-mass index: underweight (<18.5), normal (18.5-24.9), overweight (25.0-25.9), obese (≥30.0); **LT**, liver transplantation; **AIH**, autoimmune hepatitis; **PSC**, primary sclerosing cholangitis; **PBC**, primary biliary cholangitis; **OR**, odds ratio; **CI**, confidence interval; **NA**, not available due to low number of cases; **aOR**, odds ratio adjusted for age, sex, smoking, and BMI.
